# ERRγ target genes are poor prognostic factors in Tamoxifen-treated breast cancer

**DOI:** 10.1186/s13046-015-0150-9

**Published:** 2015-05-15

**Authors:** Subha Madhavan, Yuriy Gusev, Salendra Singh, Rebecca B Riggins

**Affiliations:** Department of Oncology, Lombardi Comprehensive Cancer Center, Georgetown University Medical Center, Washington DC, 20057 USA

**Keywords:** Estrogen-related receptor gamma, Tamoxifen, ER+ breast cancer, MAPK, Apoptosis

## Abstract

**Background:**

One-third of estrogen (ER+) and/or progesterone receptor-positive (PGR+) breast tumors treated with Tamoxifen (TAM) do not respond to initial treatment, and the remaining 70% are at risk to relapse in the future. Estrogen-related receptor gamma (ESRRG, ERRγ) is an orphan nuclear receptor with broad, structural similarities to classical ER that is widely implicated in the transcriptional regulation of energy homeostasis. We have previously demonstrated that ERRγ induces resistance to TAM in ER+ breast cancer models, and that the receptor’s transcriptional activity is modified by activation of the ERK/MAPK pathway. We hypothesize that hyper-activation or over-expression of ERRγ induces a pro-survival transcriptional program that impairs the ability of TAM to inhibit the growth of ER+ breast cancer. The goal of the present study is to determine whether ERRγ target genes are associated with reduced distant metastasis-free survival (DMFS) in ER+ breast cancer treated with TAM.

**Methods:**

Raw gene expression data was obtained from 3 publicly available breast cancer clinical studies of women with ER+ breast cancer who received TAM as their sole endocrine therapy. ERRγ target genes were selected from 2 studies that published validated chromatin immunoprecipitation (ChIP) analyses of ERRγ promoter occupancy. Kaplan-Meier estimation was used to determine the association of ERRγ target genes with DMFS, and selected genes were validated in ER+, MCF7 breast cancer cells that express exogenous ERRγ.

**Results:**

Thirty-seven validated receptor target genes were statistically significantly altered in women who experienced a DM within 5 years, and could classify several independent studies into poor vs. good DMFS. Two genes (EEF1A2 and PPIF) could similarly separate ER+, TAM-treated breast tumors by DMFS, and their protein levels were measured in an ER+ breast cancer cell line model with exogenous ERRγ. Finally, expression of ERRγ and these two target genes are elevated in models of ER+ breast cancer with hyperactivation of ERK/MAPK.

**Conclusions:**

ERRγ signaling is associated with poor DMFS in ER+, TAM-treated breast cancer, and ESRRG, EEF1A2, and PPIF comprise a 3-gene signaling node that may contribute to TAM resistance in the context of an active ERK/MAPK pathway.

## Background

With an estimated 1.38 million new cases diagnosed annually, breast cancer is a global public health challenge [1]. Endocrine therapy administered as an antiestrogen, such as Tamoxifen (TAM) or Fulvestrant, or an aromatase inhibitor (AI), such as Letrozole, Anastrozole, or Exemestane, is the least toxic and most effective means by which to manage hormone-dependent breast cancers. TAM increases overall survival from invasive breast cancer, reduces the incidence of estrogen receptor-α positive (ER+) disease in high-risk women, and can reduce the rate of postmenopausal osteoporotic bone loss [2,3]. It remains the standard of care for pre-menopausal breast cancer. When compared to adjuvant TAM in post-menopausal women, AIs alone or in sequence with TAM show significantly improved disease-free survival [4,5], while only letrozole provides a corresponding improvement in overall survival [6]. Thus, the optimal endocrine therapy regimen – and appropriate length of treatment - remains controversial for post-menopausal women.

Whichever way these controversies are resolved, both AIs and TAM will remain as key modalities in the management of ER+ breast cancers. Unfortunately, the inability of endocrine therapies to cure many women with ER+ disease will also remain. For example, one-third of ER+/progesterone receptor-positive (PGR+) breast tumors treated with TAM do not respond to initial treatment, and the remaining 70% are at risk to relapse in the future [7,8]. The development of resistance to AIs is also clearly documented [9-12]. A number of mechanisms have been proposed to regulate antiestrogen or TAM resistance in ER+ breast cancer, including changes in the expression or activity of genes and proteins that regulate tumor cell survival [13,14]. These tend to fall into 3 broad categories: genes that are (or can be) direct transcriptional targets of ER (e.g. PGR, CCND1, MYC); genes that are co-regulators which directly bind to ER or act on ER to modify its function (e.g. AKT, MAPK, AIB1, XBP1), and genes that can also act functionally independent of the estrogen receptor (e.g. BCAR1, BCAR3, IRF1). The identification of microRNA [15] and long non-coding RNA [16] signatures with prognostic power in ER+ breast cancers has further enhanced our understanding of receptor-driven signaling.

Estrogen-related receptor gamma (ESRRG, ERRγ) is an orphan nuclear receptor with broad, structural similarities to classical ER that is widely implicated in the transcriptional regulation of energy homeostasis [17]; in breast cancer, ERRγ is preferentially expressed in ER+ disease [18]. We have previously published that *a*. ERRγ is upregulated during the acquisition of Tamoxifen (TAM) resistance by ER+ breast cancer cells and *b*. overexpression of ERRγ is sufficient to induce TAM resistance [19], and *c*. ERRγ’s transcriptional activity and ability to induce TAM resistance is enhanced by activation of the ERK/MAPK pathway [20]. ERRγ overexpression also induces proliferation in ER+ breast cancer cells in the presence or absence of estrogen [21], and cooperates with cytoplasmic proline, glutamic acid and leucine rich protein 1 (PELP1) to inhibit TAM-mediated death in normal human mammary epithelial cells [22]. Interestingly expression of ESRRG is also significantly associated with a reduction in pathologic complete response (pCR) in locally advanced breast tumors treated with chemotherapy [23]. Our central hypothesis is that hyper-activation or over-expression of ERRγ induces a pro-survival transcriptional program that impairs the ability of TAM to inhibit the growth of ER+ breast cancer.

One of the barriers faced in addressing this hypothesis is translating data from laboratory/cell line studies into meaningful observations in breast cancer clinical data. For example, in one clinical study we found that ERRγ mRNA is significantly overexpressed in surgical (*i.e*. pre-treatment) ER+ breast tumor specimens from women who relapsed while receiving TAM [24], but this result is either not observed or not statistically significant in several other publicly available datasets of TAM-treated, ER+ breast cancer patients. Chang et al. [25] reported similar challenges in correlating mRNA expression of family member ERRα with poor outcome in breast cancer clinical specimens, but successfully generated a reproducible measure of ERRα activity *in vivo* by monitoring the expression of receptor target genes. We therefore examined the expression of validated ERRγ target genes in publicly available ER+ breast cancer datasets as a proxy for receptor activity rather than expression, which we propose is similarly a more relevant measure of *in vivo* ERRγ function in endocrine therapy response and resistance.

## Methods

### ERRγ target gene selection

Genes were selected from two independent studies in which chromatin immunoprecipitation (ChIP) for ERRγ was performed, and target genes were subsequently validated. Dufour *et al*. [26] performed high-throughput ChIP-on-chip on wild type adult mouse heart tissues, while Eichner *et al*. [27] analyzed ERRγ chromatin binding by conventional ChIP in BT-474 human breast cancer cells.

### Clinical datasets, gene expression analyses and functional annotation

Raw data from three publicly available datasets containing ER+ breast tumor surgical specimens were downloaded from Gene Expression Omnibus: Loi et al. GSE6532 and GSE91915, [28]; Zhou et al. GSE7378, [29]; and Zhang, GSE12093, [30]. Data processing pipelines in G-DOC [31] were used to obtain lists of differentially expressed genes (DEGs; fold change ≥1.5 and uncorrected p ≤ 0.05) from all datasets as described in [32].

### Cell culture, expression constructs, transfection, western blot analysis, and cell line datasets

MCF7 cells were originally provided by Marvin Rich (Karmanos Cancer Institute, Detroit, MI, USA), and cultured in improved minimal essential media (IMEM) supplemented with 5% fetal bovine serum (FBS). The pSG5 plasmid vector with cDNA insert encoding wild type, hemagglutinin (HA)-tagged murine ERRγ (100% identical to human ERRγ) has been described previously [19,20,33]. MCF7 cells were transiently transfected with HA-ERRγ or pSG5 empty vector for 27 h using JetPrime (VWR, Radnor, PA, USA) prior to whole cell lysis, polyacrylamide gel electrophoresis, protein transfer to nitrocellulose membranes, immunoblotting, and chemiluminescent detection performed as described in [20,34]. Primary antibodies used were: anti-HA.11 clone 16B12 at 1:500 (Covance, Princeton, NJ, USA); anti-EEF1A2 SAB2100650 at 1:500 (Sigma, St. Louis, MO, USA); and anti-PPIF SAB4500035 at 1:500 (Sigma). Membranes were reprobed for β-actin (Sigma, 1:10,000) as a loading control. NIH ImageJ (http://rsbweb.nih.gov/ij/) was used for densitometric analysis of ERRγ (HA), EEF1A2, and PPIF expression relative to β-actin. Levels of ESRRG, EEF1A2, and PPIF mRNA in MCF7 and BT-474 cell line samples published in [35,36] were obtained from ONCOMINE [37].

### Statistical analysis

The KM Plotter Tool (http://kmplot.com/analysis/) [38] was used to calculate hazard ratios, confidence intervals, and log-rank *P* values for the aggregated breast cancer clinical studies. All other statistical analyses were performed in GraphPad Prism 5.0c for Mac (GraphPad Software, Inc., La Jolla, CA, USA) using the Mantel-Cox log-rank test, *χ*^2^ test, or Mann Whitney rank sum test, as indicated. Statistical significance is defined as *P* ≤ 0.05.

## Results and discussion

### Identification of ERRγ target genes

ERRγ can stimulate transcription from multiple DNA response elements: the palindromic estrogen response element (ERE), a half site known as the estrogen-related receptor response element (ERRE) which it shares with other orphan nuclear receptors (e.g. steroidogenic factor 1 response element, SF1RE), and indirectly through either the activator protein 1 (AP1) (reviewed in [24]) or the specificity protein 1 (SP1) response element [39]. In addition, Deblois *et al*. identified the hybrid element ERRE/ERE as the major binding site for another member of the estrogen-related receptor family (ERRα) in breast cancer [40], which we have recently demonstrated can also be regulated by ERRγ [20]. However, the most comprehensive collection of validated ERRγ transcriptional targets comes from two independent, published studies in which high-throughput chromatin immunoprecipitation (ChIP-on-chip), or standard ChIP, data were obtained for ERRγ binding to the ERRE half site in promoters/upstream regulatory regions (Table 1). Gene IDs identified in [26] were converted from *Mus musculus* to *Homo sapiens* nomenclature using Pathway Studio, then merged with those from [27].

**Table 1:** **Sources of ERRγ target genes**

**Study [Reference]**	**Study type**	**Species**	**Tissue**	**# ERRγ targets**
Dufour et al. [26]	ChIP-on-chip	mouse	adult heart	231 validated
Eichner et al. [27]	Standard ChIP	human	BT-474 human breast cancer cell line	15 validated

**Figure 1:**
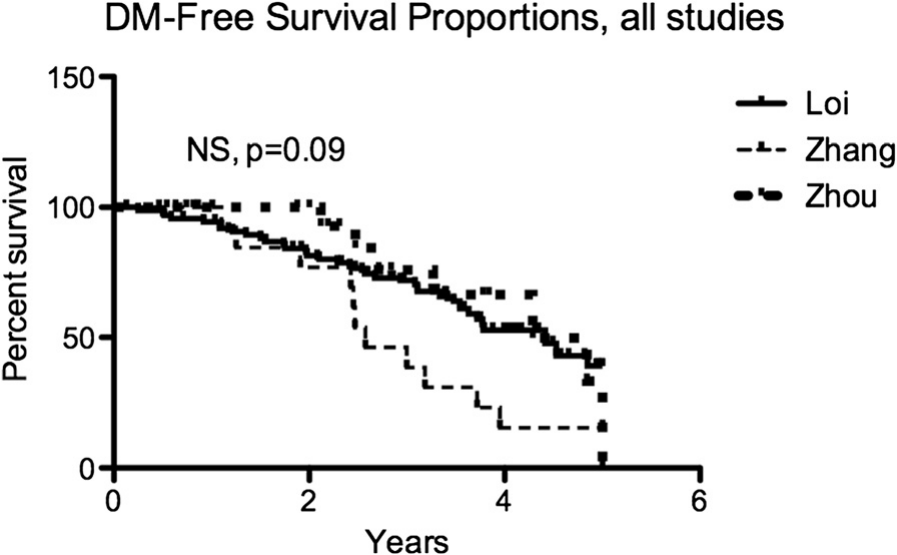
Selected clinical studies have similar proportions of distant metastasis-free survival (DMFS). Survival data were plotted using the Kaplan-Meier estimator, which show non-significant differences in DMFS data amongst the 3 studies. Log-rank p = 0.09.

We used the Georgetown Database of Cancer (G-DOC, [31]) to examine the expression of these ERRγ target genes in TAM-resistant and –responsive human breast tumors from three independent clinical datasets (Table 2). Data are derived from ER+ breast tumor specimens collected at the time of surgery, prior to initiation of TAM therapy. We selected these three datasets because they *a)* are primarily comprised of ER+ breast cancer patients who received TAM as their only systemic therapy (with the exception of 18 patients in Zhou), *b)* utilize compatible expression array platforms, and *c)* have sufficient length of follow-up to perform a meaningful comparison between those patients with documented distant metastasis ≤5 years (Event, 5yrE) and those with no distant metastasis ≤5 years (Censored, 5yrC). All 3 studies have similar overall distant metastasis (DM)-free survival proportions (Figure 1). We also, where available, examined 3 clinical parameters that could introduce bias into our results, because each has independently been shown to be a prognostic factor for DM [41]: age, lymph node status, and primary tumor size. While primary tumor size is not significantly different between groups, patients in the 5yrE group (documented DM) are significantly younger (Mann Whitney rank sum test, p < 0.01) and more likely to be lymph node-positive (*χ*^2^, p < 0.0001) than those in the 5yrC group (no documented DM within 5 years).

**Table 2:** **Breast cancer datasets**

**Study [Reference]**	**Study type**	**Sample type**	**Array platform(s)**	**GSE accession #**
Loi et al. [28]	Microarray	Surgical specimen	U133A, B, Plus 2.0	GSE6532
Loi et al. [28]	Microarray	Surgical specimen	U133A, B, Plus 2.0	GSE9195
Zhou et al. [29]	Microarray	Surgical specimen	U133A	GSE7378
Zhang et al. [30]	Microarray	Surgical specimen	U133A	GSE12093

### ERRγ target genes in TAM-resistant breast tumors

**Figure 2:**
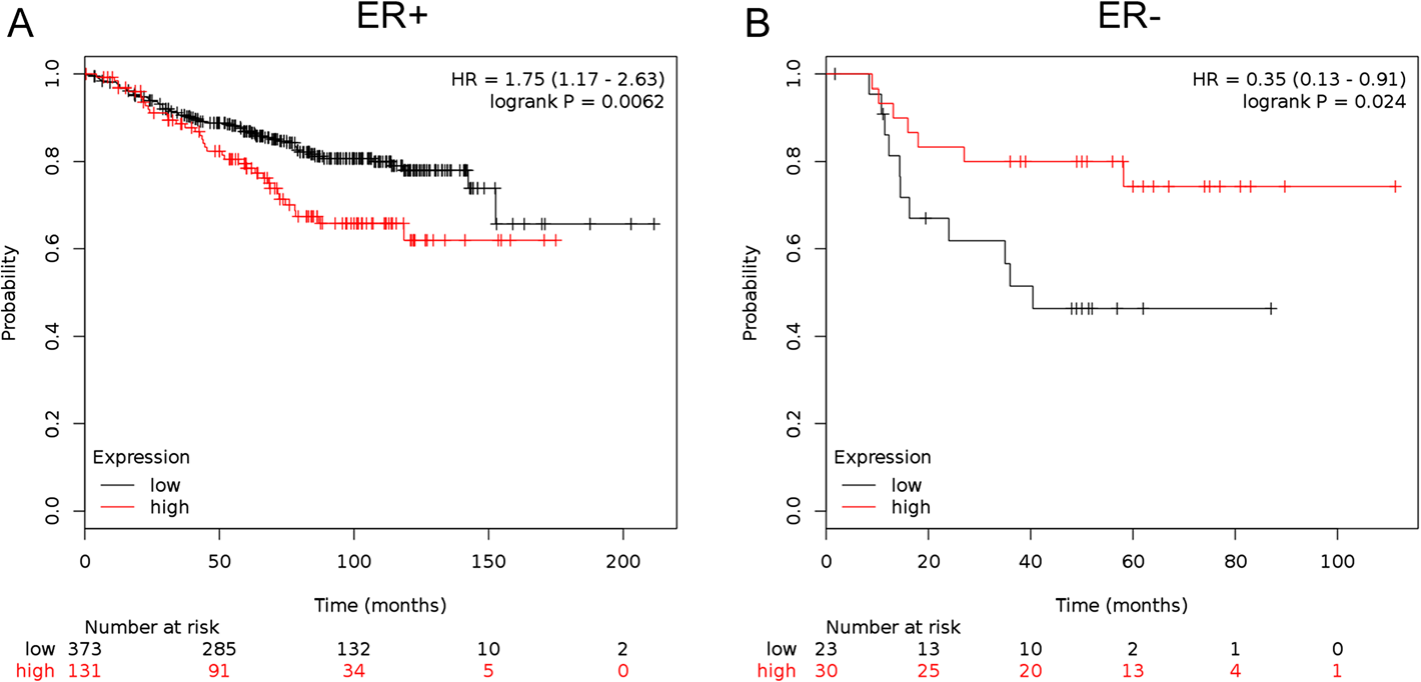
ERRγ and its target genes predict poor DMFS in ER+, but not ER-, breast cancer. Gene symbols for 35 of the 37 ERR target DEGs (2 were not annotated) were entered into KM Plotter and used to classify DMFS data from women with ER+, TAM-treated breast cancer (**A**, n = 504, HR = 1.75, log-rank p = 0.006) or ER-, chemotherapy-treated breast cancer (**B**, n = 53, HR = 0.35, log-rank p = 0.024).

We next identified statistically significant, differentially expressed genes (DEGs; fold change ≥1.5 and uncorrected p ≤ 0.05) in the 5yrE group for each of the studies, and overlaid these lists with those of the validated ERRγ targets. Then, to ensure that these ERRγ targets were predictive of TAM-resistant distant metastasis rather than simply poor prognostic factors, we excluded from the list genes that showed the same regulation (direction of fold change) in an independent dataset of ER+, lymph node-negative breast cancer patients from (GSE7390, [42]) who received no systemic therapy. This resulted in a final list of 37 DEGs (32 up-regulated, 5 down-regulated; Table 3). Using the KM Plotter Tool (http://kmplot.com/analysis/) [38], we showed that these 37 DEGs (alone or with the addition of ESRRG) serve as a molecular signature that is significantly associated with poor distant metastasis-free survival (DMFS) in 504 women with ER+ breast cancer treated with TAM monotherapy (Figure 2A, HR = 1.75, p = 0.0065). By contrast, these 37 DEGs show the opposite association (i.e. with improved DMFS) in 53 women with ER- breast cancer treated with chemotherapy (Figure 2B, HR = 0.35, p = 0.024).

**Table 3:** **37 DEGs significantly associated with distant metastasis (DM) in ER+, TAM-treated patients**

**Analysis:**	**DMFS ≤5 years Censor vs. Event**		
**Treatment:**	**TAM**	**TAM**	**TAM+**
**Patient #:**	**95 vs. 68**	**3 vs. 11**	**17 vs 7**
**Gene**	**Loi**	**Zhang**	**Zhou**
ACADM		up	
**AHSA1**	up		up
ARIH2			up
ATP5C1		up	
ATP5F1		up	
CENPT			up
CSMD1	down		
DLST			up
EEF1A2			up
ETFB	up		
GTPBP4		up	
HSPA9	up		
IDH1		up	
MED23			up
MYCN	down		
**NADK**	up		up
NDUFA8		up	
NDUFB5		up	
NDUFS1		up	
NDUFS7	up		
ORMDL1	up		
PAN2			up
PCMTD2			up
PPIF		up	
PTCD3			up
PTPN18		down	
RAB11B		down	
**RAB21**		up	up
*RARA*	down		
SDHD		up	
SLC35E2			up
*SPTLC2*	up		
**SUCLA2**		up	
TIMM17A		up	
TRRAP			up
TSPAN8			up
UNC50		up	

Legend: *italicized*, also present in prognostic list, but opposite regulation; **bold**, present in >1 dataset.

### ERRγ target gene functional annotation and validation

**Figure 3:**
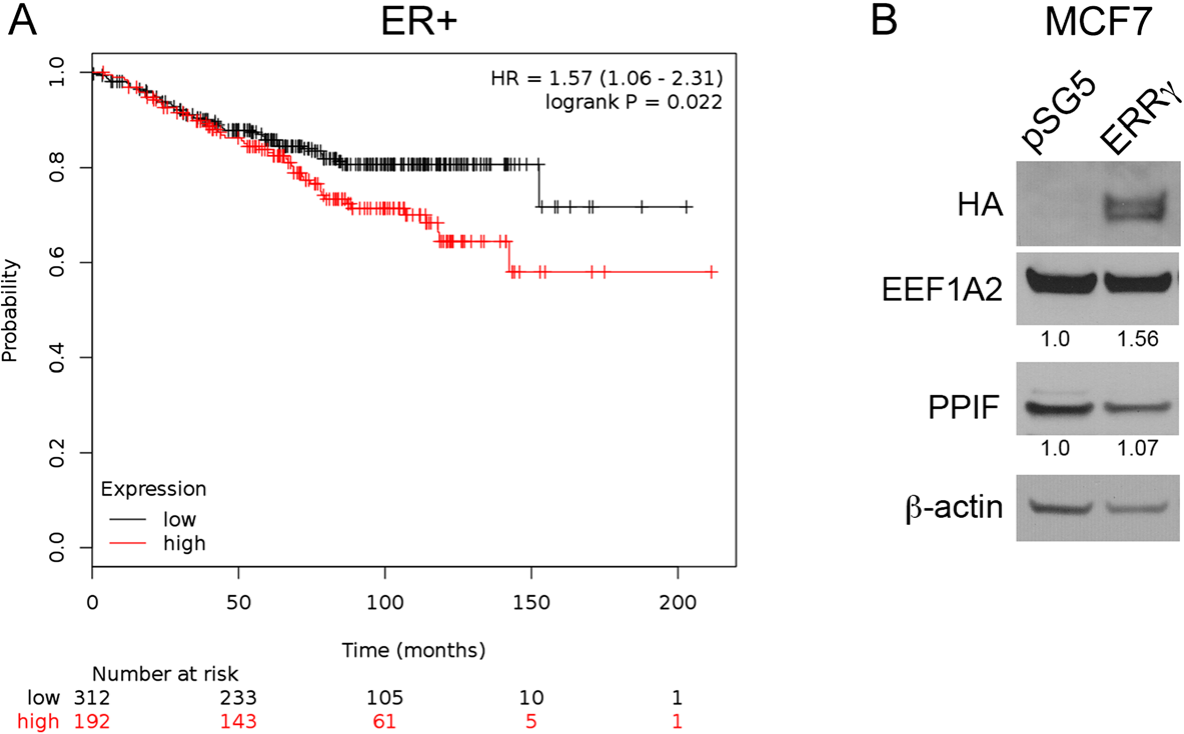
Predictive value and expression of ERRγ target genes EEF1A2 and PPIF in ER+ breast cancer. **A**, The 3-gene signature of ESRRG, EEF1A2, and PPIF predicts poor DMFS in ER+, TAM-treated breast cancer using KM Plotter (n = 504, HR = 1.57, log-rank p = 0.022). **B**, Expression of EEF1A2 and PPIF protein in MCF7 cells transiently expressing exogenous hemagglutinin (HA-) tagged ERRγ. β-actin serves as the loading control. Relative expression of EEF1A2 (1.57) and PPIF (1.07) in ERRγ-transfected cells vs. empty vector control (1.0) was analyzed using NIH Image J.

Using Gene Set Enrichment Analysis tools in Pathway Studio, the Molecular Signatures Database (MSigDB, [43]), and WebGestalt [44], we examined the ERRγ gene signature for commonalities in Gene Ontology and functional annotations. Given that ERRγ and its family members promote mitochondrial biogenesis and control the transcription of nearly all essential enzymes of the oxidative phosphorylation pathway [17], it is not surprising that many genes associated with respiratory oxidative phosphorylation (p = 0.00024) and the electron transport chain (p = 2.18E-13) are significantly overrepresented. However, other functional categories highly relevant to the TAM-resistant phenotype are also enriched, including apoptosis (p = 0.027), protein folding (p = 0.0023) and mitochondrial protein transport (p = 5.88E-05). Two novel and particularly interesting ERRγ target genes in this regard are eukaryotic elongation factor 1A2 (EEF1A2), a putative oncogene and elongation factor that delivers tRNAs to active ribosomes, and peptidylprolyl isomerase factor F (PPIF, more commonly known as Cyclophilin D), a key component of the mitochondrial protein folding machinery and the inner membrane permeability transition pore. EEF1A2 strongly promotes cancer cell proliferation and resistance to apoptosis in several malignancies [45-47]. The role of PPIF in apoptosis is less clear; many studies describe a pro-death role for PPIF and the mitochondrial permeability transition pore in general, while others [48] show that PPIF can suppress apoptosis induced by exogenous stimuli such as TNF, UV radiation, or arsenic trioxide, possibly through negative regulatory interactions with Bcl-2 [49,50]. In addition, a three-gene signature comprised of ERRγ, EEF1A2, and PPIF alone is significantly associated with poor distant metastasis-free survival (DMFS) in the same 504 women with ER+ breast cancer who received Tamoxifen monotherapy (Figure 3A, HR = 1.57, p = 0.022). To validate EEF1A2 and PPIF as target genes of ERRγ in ER+ breast cancer, we measured their protein expression in MCF7 cells transiently transfected with ERRγ, which we [20] and others [21] have shown induces Tamoxifen resistance and estrogen-independent growth, respectively (Figure 3B). While PPIF is not induced, EEF1A2 protein is ~1.5-fold increased in cells transfected with ERRγ relative to the β-actin loading control.

**Figure 4:**
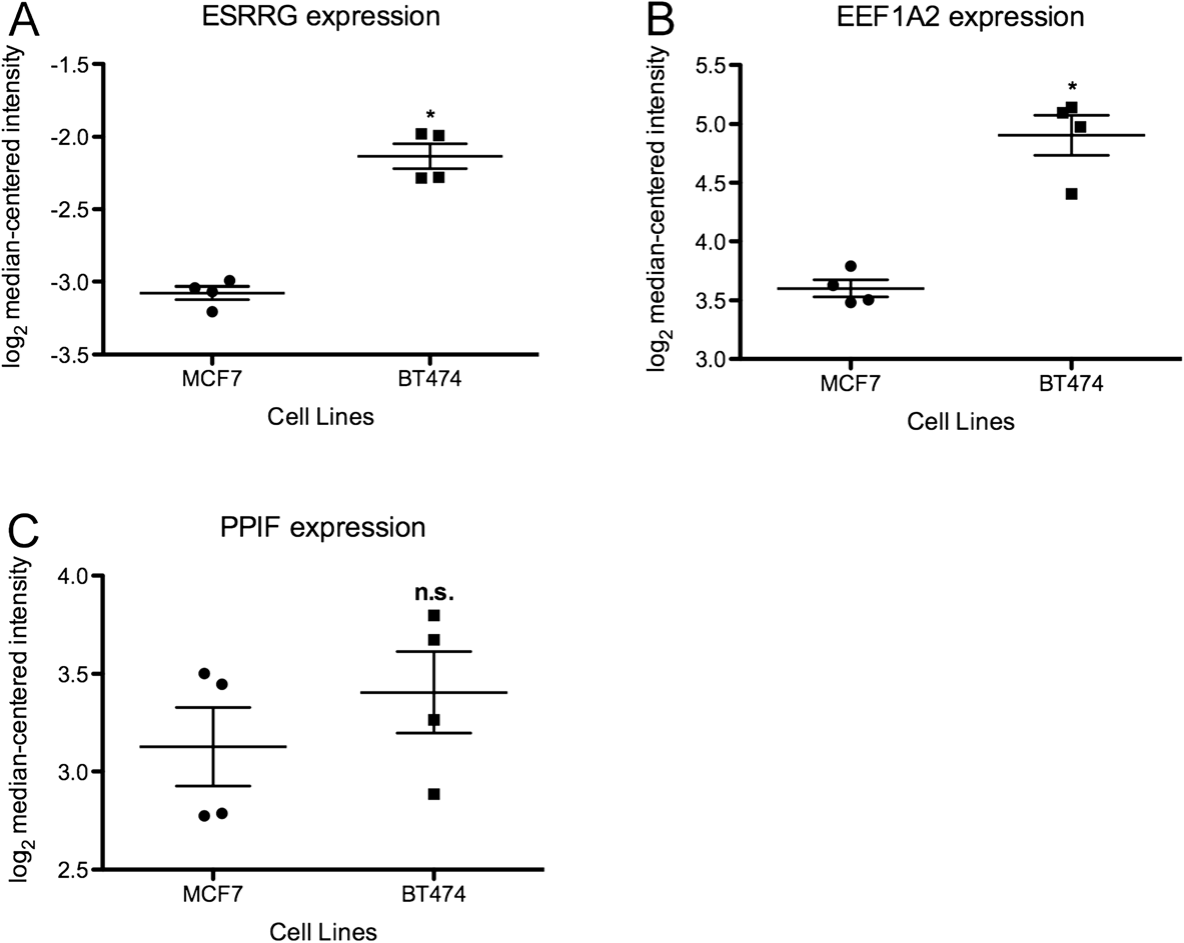
Expression of ESRRG, EEF1A2, and PPIF correlates with ERK/MAPK activation status in ER+ breast cancer cells. Gene expression data from Rae et al. obtained from ONCOMINE were analyzed for ESRRG **(A)**, EEF1A2 **(B)**, and PPIF **(C)** in n = 3 replicates per cell line. Mann Whitney rank-sum p < 0.05 (*).

We have recently shown that regulation of ERRγ protein by ERK/MAPK enhances the receptor’s transcriptional activity and is required for its ability to induce Tamoxifen resistance in ER+ breast cancer cells [20,51]. However, the relationship between ERK/MAPK and either ERRγ or EEF1A2 at the mRNA level has not been characterized. We therefore examined their message levels in gene expression microarray data from ER+ MCF7 and BT474 breast cancer cells [35], the latter well known for overexpression of HER2 and hyperactivation of the ERK/MAPK pathway. Expression of ESRRG (Figure 4A) and EEF1A2 (Figure 4B), but not PPIF (Figure 4C), is significantly higher in BT474 cells. Interestingly in a second dataset [36], EEF1A2 expression is markedly and significantly induced in ER+ MCF7 breast cancer cells in which either MEK (4.01-fold increase, p = 0.027) or HER2 (3.34-fold increase, p = 0.036) has been exogenously expressed vs. the empty vector control. PPIF is also modestly induced (MEK: 1.38-fold increase, p = 0.01; HER2: 1.27-fold increase, p = 0.007).

## Conclusions

The goal of the present study was to determine whether ERRγ target genes are associated with reduced DMFS in ER+ breast cancer treated with Tamoxifen. Our findings suggest that i. ERRγ signaling is associated with poor DMFS in ER+, TAM-treated breast cancer, and ii. ESRRG, EEF1A2, and PPIF comprise a 3-gene signaling node that may contribute to Tamoxifen resistance in the context of an active ERK/MAPK pathway.

## Abbreviations

TAM: Tamoxifen
AI: Aromatase inhibitor
ER+: Estrogen receptor-positive
PGR+: Progesterone receptor-positive
CCND1: Cyclin D1
MAPK: Mitogen-activated protein kinase
AIB1: Amplified in breast cancer 1
XBP1: X-box binding protein 1
BCAR1: Breast cancer antiestrogen resistance 1
BCAR3: Breast cancer antiestrogen resistance 3
IRF1: Interferon regulatory factor 1
ESRRG or ERRγ: Estrogen-related receptor gamma
ERK: Extracellular signal-regulated kinase
ChIP: Chromatin immunoprecipitation
HA: Hemagglutinin
EEF1A2: Eukaryotic elongation factor 1A2
PPIF: Peptidylprolyl isomerase factor F
KM: Kaplan-Meier
ERE: Estrogen response element
ERRE: Estrogen-related response element
AP1: Activator protein 1
SP1: Specificity protein 1
G-DOC: Georgetown Database of Cancer
5yrE: Event at 5 years
5yrC: Censored at 5 years
DM: Distant metastasis
DEGs: Differentially expressed genes
DMFS: Distant metastasis-free survival
HR: Hazard ratio
MSigDB: Molecular Signatures Database
TNF: Tumor necrosis factor
HER2: Human epidermal growth factor receptor 2
